# Supplementary material for: The interactome of intact mitochondria by cross-linking mass spectrometry provides evidence for coexisting respiratory supercomplexes
Source: Mol Cell Proteomics. 2017 Dec 8;17(2):216–32. doi: 10.1074/mcp.RA117.000470 (PMC5795388; doi:10.1074/mcp.RA117.000470)
Supplement: Supplemental Data [file supp_RA117.000470_133922_0_supp_23978_fzffwf.zip › spectra_annotation/mito_DR_spectra_annotation/498-1-9-1-3-1.pdf]

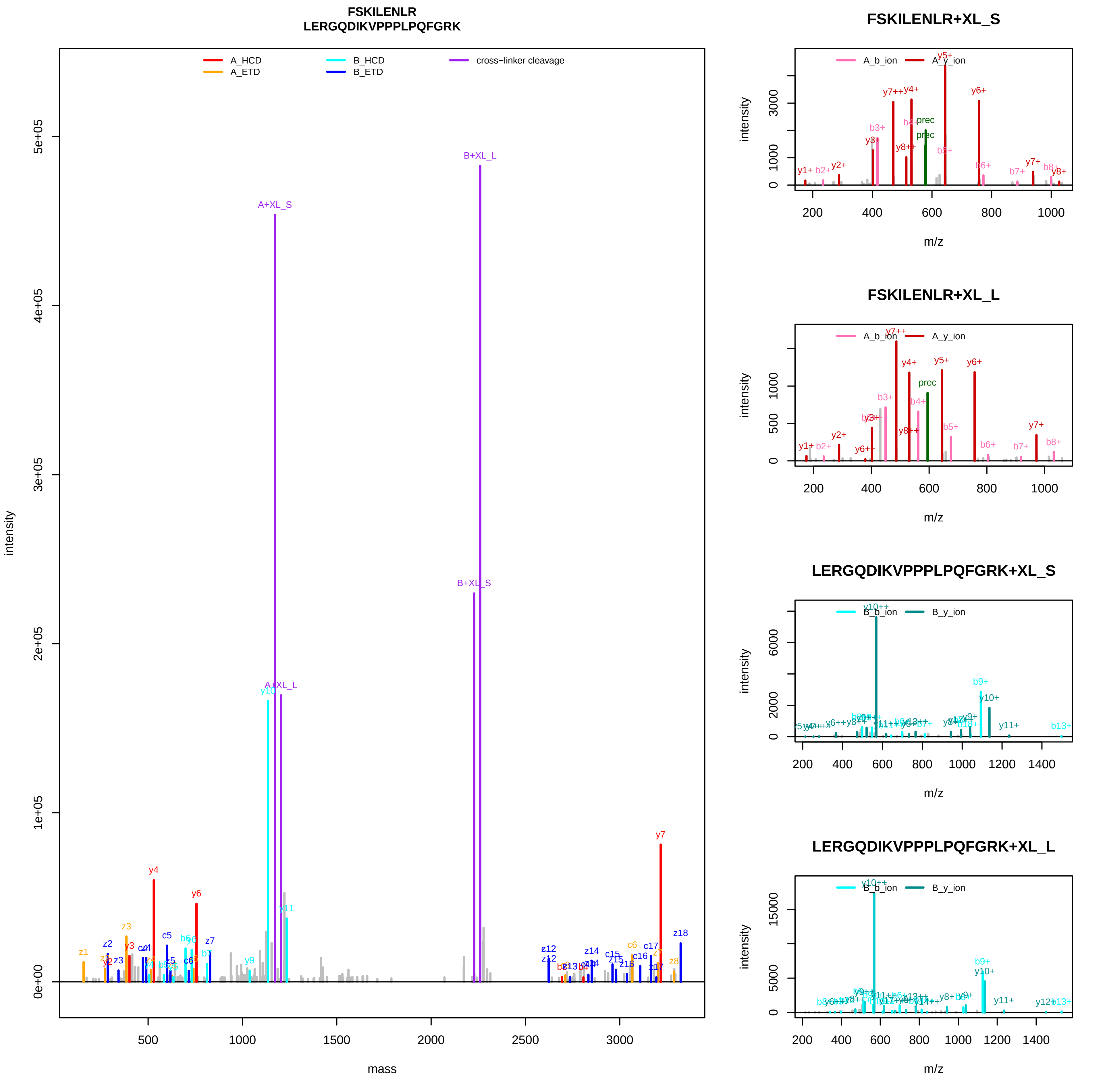
FSKILENLR
LERGQDIKVPPPLPQFGRK
A_HCD
A_ETD
B_HCD
B_ETD
cross−linker cleavage
intensity
mass
0e+00
1e+05
2e+05
3e+05
4e+05
5e+05
500
1000
1500
2000
2500
3000
A+XL_S
B+XL_L
B+XL_S
A+XL_L
y10
y11
y4
y6
y7
y3
y9
z1
z2
z3
c5
c6
c16
c17
z7
z8
z12
z14
z15
z16
z18
FSKILENLR+XL_S
FSKILENLR+XL_L
LERGQDIKVPPPLPQFGRK+XL_S
LERGQDIKVPPPLPQFGRK+XL_L
A_b_ion
A_y_ion
B_b_ion
B_y_ion
prec
m/z
y5+
y7++
y4+
y6+
y3+
b3+
b4+
y8++
y2+
y1+
b2+
b5+
b6+
b7+
y7+
b8+
y8+
y10++
b9+
y10+
y11+
b13+
y12+
y13+
0
1000
3000
500
2000
6000
5000
15000
200
400
600
800
1000
1200
1400
